# Gastronomical Delight: Micronutrients Protect against Arsenic Lesions

**Published:** 2008-08

**Authors:** M. Nathaniel Mead

Studies in South Asia suggest that antioxidants may mediate many of the dermatologic symptoms associated with exposure to arsenic in drinking water. Nonetheless, the mitigating effects of diet on arsenic-related premalignant skin lesions are largely unknown, particularly in the context of the typical Bangladeshi diet. A new cross-sectional study using baseline data from the Health Effects of Arsenic Longitudinal Study (HEALS), 2000–2002, is the first systematic, population-based attempt to assess the association between micronutrient intake and the prevalence of arsenic-induced skin lesions **[*EHP* 116:1056–1062; Zablotska et al.]**.

As many as a third of the people living in Bangladesh have been exposed to arsenic-tainted water levels above the national limit of 50 ppb, with many levels as high as 800 ppb. Several studies have shown an association between drinking arsenic-rich water and development of skin lesions, a common outward sign of chronic arsenic exposure.

HEALS is a population-based prospective cohort study in Araihazar, Bangladesh, using individual-level time-weighted measures of arsenic exposure via drinking water. The present study relied on detailed daily diet information obtained from all participants using a food frequency questionnaire along with U.S. Department of Agriculture nutritional tables. The analyses were aimed at clarifying the effects of the B vitamin group and antioxidants (vitamins A, C, and E) on the risk of arsenic-related skin lesions. Because supplements and food fortification are rare in Bangladesh, only dietary intakes of these micronutrients were considered.

Skin lesions were identified among 10,628 subjects according to a structured clinical protocol during screening that was confirmed with further clinical review. Dietary intake of B_1_, B_6_, and B_9_ and all three antioxidants significantly reduced the risk of arsenic-related skin lesions. For example, for individuals with the highest vitamin intake, the risk of arsenic-induced skin lesions was significantly reduced by 60% for vitamin E.

The investigators conclude that intakes of B vitamins and antioxidants at doses greater than the current recommended daily amounts for Bangladesh might lower the risk of arsenic-related skin lesions. However, the research team observed that there was a high prevalence of micronutrient deficiency in Bangladesh, with the potential protective modifying effects of these vitamins restricted to the medium and upper consumption levels. Public health measures to assist this population may need to include either supplementation or food fortification to achieve a significant degree of protection from chronic arsenic exposures.

## Figures and Tables

**Figure:**
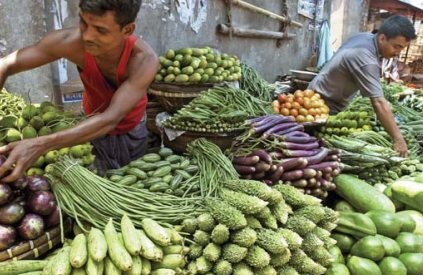
A diet rich in B vitamins and antioxidants may counter some of the effects of chronic arsenic ingestion

